# Cochrane corner: digital contact tracing technologies in epidemics

**DOI:** 10.11604/pamj.supp.2020.37.1.25986

**Published:** 2020-09-12

**Authors:** Anelisa Jaca, Chinwe Juliana Iwu, Charles Shey Wiysonge

**Affiliations:** 1 Cochrane South Africa, South African Medical Research Council, Cape Town, South Africa,; 2 Department of Nursing and Midwifery, Faculty of Medicine and Health Sciences, Stellenbosch University, Cape Town, South Africa,; 3 School of Public Health and Family Medicine, University of Cape Town, Cape Town, South Africa,; 4 Department of Global Health, Faculty of Medicine and Health Sciences, Stellenbosch University, Cape Town, South Africa

**Keywords:** Contact tracing, digital tracing, infectious diseases

## Abstract

Contact tracing is a public health measure implemented to control the spread and break the chains of transmission of an infectious disease. It is done by identifying, assessing, and managing people who have been exposed to an infectious disease to prevent onward transmission. We summarize findings from a rapid Cochrane review that included cohort and modelling studies to assess the benefits and harms of digital solutions for identifying contacts of confirmed positive cases of an infectious disease. The review included 12 studies, which assessed digital contact tracing for the following infectious diseases: Ebola, tuberculosis, pertussis and coronavirus disease 2019 (COVID-19). This review revealed low-certainty evidence of a decrease in secondary cases of the targeted infectious disease, if digital contact tracing was used. However, it is uncertain from the currently available evidence whether digital contact tracing would produce more reliable counts of contacts and reduce the time taken to complete contact tracing. Therefore, implementation of digital contact tracing in the context of the ongoing coronavirus pandemic in African countries should be accompanied by a robust monitoring and evaluation framework. There should be an evaluation and documentation of the benefits, cost-effectiveness, acceptability, feasibility, equity impacts, and unintended consequences of the intervention.

## Commentary

COVID-19 is caused by the severe acute respiratory syndrome-coronavirus-2 (SARS-CoV-2), and spreads from person-to-person through droplet and contact transmission [1]. The number of confirmed COVID-19 infections in Africa are rising as different countries in this region report new cases every day. The Africa Centres for Disease Control and Prevention, Africa has registered 1,307,444 cases and 31,520 deaths [2]. These number of cases are reported as countries continue to reopen their economies slowly after the shutdowns [2]. To control the spread of COVID-19, interventions are required to interrupt the chains of transmission to ensure that the number of new cases are kept below 1 (effective reproduction number < 1) [3]. Strategies, namely, case identification, isolation, testing and care, and contact tracing and quarantine, are vital actions needed to decrease transmission and control the epidemic [3]. Contact tracing for COVID-19 requires identifying individuals who may have been exposed to COVID-19 and following them up every day for 14 days. For contact tracing to be effective, countries must have sufficient capacity to test suspect cases in on time [3]. However, if this not possible, testing and contact tracing strategies may instead focus on specific high-risk areas with susceptible people, such as hospitals, care homes, or any other closed surroundings [3].

Tracing individuals who have been in contact with infected people can be effective in limiting the spread of an infection and number of deaths [4]. Implementing contact tracing helps in delaying the effects and impact of the pandemic by providing more time for preparedness and response efforts and reducing the number of people who are exposed and infected [5]. Decreasing the number of people who are infected means that fewer people will get sick or die, and that health systems will not be overwhelmed, i.e., hospitals and doctors will be better able to take care of the sick [6]. Various methods of contact tracing including traditional or manual contact tracing, self-reported diaries and surveys, interviews, other standard methods for determining close contacts, and other technologies compared to digital solutions can be used to achieve this, Digital contact tracing is a method of contact tracing relying on tracking systems, most often based on mobile devices, to determine contact between an infected patient and a user. It came to public prominence in the form of COVID-19 apps during the COVID-19 pandemic [7]. Numerous countries in Africa have either implemented or are considering the use digital contact-tracing as one of their strategies for controlling the spread of COVID-19. Countries like South Africa, Rwanda and Egypt have implemented digital contact tracing with the use of mobile app and cellphone tower data, respectively [8]. The World Health Organization (WHO), African region is also piloting a project with the Republic of Congo, with the aim of repurposing the Polio geographic information system (GIS) platform that was previously developed for EVD. Furthermore, WHO is also developing a global app for checking symptoms and tracing contacts, that would support Africa and other developing countries in their fight against COVID-19 [8].

In this commentary, we highlight and contextualize the findings of a rapid Cochrane review by Anglemyer and colleagues, on the benefits, harms, and acceptability of personal digital contact tracing solutions for identifying contacts of an identified positive case of an infectious disease [7]. Digital contact tracing solutions included smartphone apps, wearable devices, and hardware- and software-based solutions. These were compared to traditional or manual contact tracing methods, i.e., interviews and diaries. Outcome measures were the number of secondary cases identified from contact tracing procedures (measured with counts of secondary cases, or with the average number of secondary cases per index case); number of close contacts identified from contact tracing procedures; average length of time to complete contact tracing for a case (end point would be the last day of follow-up in the study and, if available, at seven days after case notification); acceptability and accessibility; privacy issues; and safety concerns. The review included cohort studies, cross-sectional studies, modelling studies, quasi-randomised controlled trials), randomised controlled trials (RCTs), including cluster-RCTs. The authors searched CENTRAL, Ovid MEDLINE and Embase databases from 01 January 2000 to 05 May 2020 to identify eligible studies. Two authors independently performed study selection, data extraction and risk of bias assessment [7].

This review included a total of 12 studies comprising of six cohort studies that reported quantitative data and six modelling studies that reported on modelling of digital solutions for contacting tracing [7]. Of the six cohort studies, three cohort studies were of digital solutions to contact tracing within active disease outbreak settings. One study evaluated a contact tracing app used by public health contact tracers of 18 Ebola cases in Sierra Leone over four months amid an Ebola outbreak in 2015. Contact tracing intake information collected using the app was compared to traditional paper case intake forms used by public health contact tracers of 25 cases. The app was a manual solution developed to expand paper-based contact tracing and monitoring efforts during data collection and management phases. Of the six modelling studies, four evaluated digital solutions for contact tracing in COVID-19-simulated scenarios in the UK or in nonspecific settings, while two simulated close contacts in non-specific outbreak settings [7]. There was very low-certainty evidence regarding the benefit of digital compared to manual contact tracing. Contact tracers who used an app during an Ebola outbreak, found twice as many close contacts per case compared to those who used paper forms. Likewise, some US researchers found that radio-frequency identified 45 close contacts after a pertussis outbreak relative to electronic medical records which only found 13. The reasons for low certainty of evidence in these two studies were due to issues of imprecision and serious risk of bias. There was also very low-certainty evidence that an app could decrease the time to complete a set of close contacts. The certainty of evidence for this outcome was affected by imprecision and serious risk of bias. Individuals who did contact tracing reported that digital data entry and management systems were faster to use than paper systems and possibly less prone to data loss.

Investigations conducted in low- or middle-income countries revealed some advantages of digital contact tracing systems over their manual counterparts. These studies reported that contact tracing teams found digital systems simpler to use and generally preferred them over paper systems, they were less time consuming, improved accuracy with large data, and were easier to transport compared with paper forms. However, the digital systems had challenges, for an example, there were increased costs and internet access problems compared to paper systems. Concerning privacy, devices seemed to have provided privacy regarding users who were exposed or diagnosed of the disease, although there were risks of privacy breaches from snoopers if linkage attacks occurred, particularly for wearable devices. According to modelling studies, there was also low-certainty evidence regarding a decrease in secondary cases using digital contact tracing. One study estimated an 18% reduction in in secondary cases with digital contact tracing compared to self-isolation alone, and a 35% reduction with manual contact-tracing. The certainty of evidence was due to unclear stipulations of their models, and about their assumptions of the effectiveness of manual contact tracing, where they assumed that 95% to 100% of contacts would be traced.

## Conclusion

Although modelling studies have reported that digital solutions in combination with strong public health efforts can reduce epidemics, cohort studies provide very low certainty evidence that technology can give more reliable counts of contacts and reduce the time to complete contact tracing in real epidemic situations. This therefore implies that more research needs to be conducted to provide enough evidence about the effectiveness of digital contact tracing compared to manual or paper-based methods. In addition, there needs to be more research done in the low-and-middle income regions, especially in Africa, where health systems tend to be overwhelmed when faced with disease outbreaks. Despite the limitations in certainty of evidence, digital contact tracing could potentially be useful in curbing the spread of COVID-19 and other highly infectious diseases. Governments in African countries could leverage on the existing mobile technology resources available in their countries, while taking into account, concerns around access in resource limited settings and privacy.
